# The analysis of factors increasing the odds for type 2 diabetes mellitus remission following re-do bariatric surgery after laparoscopic sleeve gastrectomy- cohort study

**DOI:** 10.1007/s00423-023-03102-0

**Published:** 2023-09-22

**Authors:** Michał Wysocki, Karol Ciszek, Justyna Rymarowicz, Piotr Zarzycki, Maciej Walędziak, Katarzyna Bartosiak, Paweł Jaworski, Wojciech Kupczyk, Jacek Szeliga, Wiesław Tarnowski, Magdalena Pisarska-Adamczyk, Piotr Małczak, Michał Pędziwiatr, Piotr Major, PROSS- Collaborative Study Group members, Tomasz Stefura, Piotr Myśliwiec, Hady Razak Hady, Paulina Głuszyńska, Monika Proczko-Stepaniak, Michał Szymański, Michał Janik, Andrzej Kwiatkowski, Magdalena Materlak, Łukasz Czyżykowski, Maciej Mawlichanów, Piotr Kowalewski, Natalia Dowgiałło-Gornowicz, Paweł Lech, Anna Harań, Grzegorz Kowalski, Rafał Mulek, Michał Kreft, Michał Orłowski, Paula Franczak, Artur Binda, Mateusz Kamiński, Maciej Pastuszka, Wojciech Lisik, Paweł Szymański, Bartosz Katkowski, Michał Leśniak

**Affiliations:** 1 Department of General Surgery and Surgical Oncology, Ludwik Rydygier Memorial Hospital, Cracow, Poland; 2 2nd Department of General Surgery, Jagiellonian University Medical College, Jakubowskiego 2 Street, 30-688 Kraków, Poland; 3 Department of General, Oncological, Metabolic and Thoracic Surgery, Military Institute of Medicine, Warsaw, Poland; 4 Centre of Postgraduate Medical Education, Orlowski Hospital, Warsaw, Poland; 5 Department of General, Gastroenterological, and Oncological Surgery, Collegium Medicum Nicolaus Copernicus University, Torun, Poland

**Keywords:** Laparoscopic sleeve gastrectomy, Type 2 diabetes mellitus, Re-do bariatric surgery, Diabetes remission, Revisional bariatric surgery

## Abstract

**Introduction:**

Metabolic/bariatric surgery is the only proven treatment for type 2 diabetes mellitus (T2D) with curative intent. However, in a number of patients, the surgery is not effective or they may experience a relapse. Those patients can be offered re-do bariatric surgery (RBS).

**Purpose:**

The study aimed to determine factors increasing the odds for T2D remission one year after RBS following primary laparoscopic sleeve gastrectomy.

**Methods:**

A multicenter retrospective cohort study was conducted between January 2010 and January 2020, which included 12 bariatric centers in Poland. The study population was divided into groups: Group 1- patients with T2D remission after RBS (*n* = 28) and Group 2- patients without T2D remission after RBS (*n* = 49). T2D remission was defined as HBA_1c_ < 6.0% without glucose-lowering pharmacotherapy and glycemia within normal range at time of follow-up that was completed 12 months after RBS.

**Results:**

Fifty seven females and 20 males were included in the study. Patients who achieved BMI < 33 kg/m^2^ after RBS and those with %EBMIL > 60.7% had an increased chance of T2D remission (OR = 3.39, 95%CI = 1.28–8.95, *p* = 0.014 and OR = 12.48, 95%CI 2.67–58.42, *p* = 0.001, respectively). Time interval between primary LSG and RBS was significantly shorter in Group 1 than in Group 2 [1 (1–4) vs. 3 (2–4) years, *p* = 0.023].

**Conclusions:**

Shorter time interval between LSG and RBS may ease remission of T2D in case of lack of remission after primary procedure. Significant excess weight loss seems to be the most crucial factor for T2D remission.

## Introduction

An excessive accumulation of adipose tissue in the body adversely affects glucose metabolism. It may lead to the development of type 2 diabetes mellitus (T2D), hypertension, and dyslipidemia which are components of metabolic syndrome [1, 2]. Metabolic surgery has replaced the term ‘bariatric surgery’ because the surgical approach is not only targeted at excess weight, but also to primarily treat type 2 diabetes (T2D) and metabolic disease [3]. Metabolic/ bariatric surgery is currently the most effective and long-lasting treatment for severe obesity [4–7]. It has a proven curative effect on T2D which cannot be achieved with pharmacological therapy [4, 7, 8]. Metabolic surgery has been shown to bring complete long-term remission of T2D and can also prevent the development of T2D. Despite its efficacy, some patients will not achieve complete remission after surgery or may experience a recurrence in a long-term follow-up. In that case, a re-do bariatric surgery (RBS) can be a viable alternative to conservative management. There is a growing number of RBS performed worldwide and in some countries, it has become the third most commonly performed bariatric procedure [9, 10]. Also in Poland their number is continuously growing [11–13]. We still lack research on the factors that are increasing odds for T2D remission one-year after RBS, following laparoscopic sleeve gastrectomy (LSG) as the primary bariatric procedure [9, 10].

## Purpose

The aim of this study is to determine which factors are increasing the odds for T2D remission one-year after RBS following primary LSG.

## Materials and methods

### Methods

A retrospective cohort study was conducted. This research did not receive any specific grant from funding agencies in the public, commercial, or not-for-profit sectors.

Patients with T2D undergoing RBS after LSG between January 2010 and January 2020 were included. An electronic database was created including patients from 12 Polish bariatric centers that entered voluntarily Polish Revision Obesity Surgery Study (PROSS) (ClinicalTrials.gov Identifier: NCT05108532). Inclusion criteria were as follow:

- age ≥ 18 years
- presence of T2D before RBS
- complete 1-year follow-up with assessment of type 2 diabetes mellitus remission.

Patients with incomplete data were excluded from the study. Each participating bariatric center provided specific data, which were processed and used in the overall analysis.

T2D remission definition was based on guidelines of American Diabetes Association (ADA), that is HBA_1c_ < 6.0% without glucose-lowering pharmacotherapy and fasting glycemia within normal range [14, 15].

Re-do bariatric surgery was defined as revision of a bariatric procedure due to inadequate weight loss or postoperative weight regain; inadequate improvement or frank recurrence of a weight-related comorbidity such as type 2 diabetes; or complications related to the primary bariatric surgery [16].

The study population was divided into two groups: Group 1 – patients with T2D remission after RBS and Group 2 – patients without T2D remission.

Each patient was qualified for surgical treatment in accordance with The Polish Guidelines for Metabolic and Bariatric Surgery [17]. All surgical procedures were performed laparoscopically in all centers [18–20].

Perioperative morbidity of re-do bariatric surgery was defined as any deviation from the standard perioperative course after RBS that required additional management within 30 days after the procedure (early morbidity), while postoperative morbidity was defined as morbidity that occurred over 30 days after RBS within the first year after RBS (late morbidity) [21]. Insufficient weight loss at participating centers was defined as achieving less than 50% EBMIL at 12 months after primary bariatric surgery. Weight regain is defined as progressive weight regain that occurs after achievement of an initial successful weight loss (defined as EBMIL > 50%) [22].

The study was designed and described according to the STROBE guidelines for observational studies [23]. The work has been reported in line with the STROCSS criteria [21].

### Surgical technique

Types of RBS in our study included: re-sleeve gastrectomy (re-SG), revisional single anastomosis sleeve ileal bypass (SASI), revisional Roux-en-Y gastric bypass (RYGB) and revisional one-anastomosis gastric bypass (OAGB).

Re-SG began with mobilization of entire sleeve from the adhesions after primary SG. A 35 F gastric tube was guided along the lesser curvature of the stomach and passed distal to the pylorus. Stapling and division was done starting 6 cm proximal to the pylorus i.e. opposite to the incisura angularis of stomach. It was done with either 4.1 or 4.2 mm cartridges (length 60 mm) depending on the thickness of stomach assessed by operator. Staple line ended at the level of left crus of diaphragm. Staple line was not routinely oversewn, treated more likely as hemostatic measure if needed.

In case of SASI, the ileocecal region was identified and a 300 cm ileal loop was measured proximally. When the pylorus and ileal loop were positioned properly a linear 45-mm stapler was used to create a sleeve ileal side-to-side anastomosis, 6 cm proximally from the pylorus. The stapler defect was closed with a running suture.

During conversion from SG to RYGB, the sleeve was typically divided just distal to the left gastric artery. Gastric pouch was created with use of linear staplers (60 mm in length). Gastric tube was 35 F for calibration. Excess gastric tissue was excised if gastric sleeve was dilated. The gastric pouch was a standard 50 ml volume. The biliopancreatic limb was 100 cm, and the Roux limb was measured for 200 cm. Gastrojejunostomy and jejunojejunostomy were performed with a linear stapled technique and wall defects were closed with running sutures. The Roux limb were ante-colic in our practice. Mesenteric defects were not routinely closed. Decision was based on operator’s practice.

During OAGB the gastric pouch was calibrated (35 F) and crafted using a linear stapler. Gastric transection was performed at the incisura angularis of the lesser curvature. In case of gastric dilatation, the excision of the excess gastric tissue was performed due to the absence of vascularization on the large curvature. The jejunal loop was lifted in an antecolic position. The length of bilopancreatic limb was 150 cm from the ligament of Treitz. Posterolateral gastrojejunostomy was performed with a linear stapler followed by continuous sewing of the staple aperture.

### Statistical analysis

Calculations were performed using STATISTICA 13.3 PL software (Tibco, CA, USA). Continuous values were presented as means with standard deviations, or medians with interquartile ranges when appropriate. Qualitative variables were compared using the Pearson χ-square with or without Yates’ correction. All available variables were analyzed in logistic regression models. *P*-values ≤ 0.05 were considered statistically significant.

## Results

**Fig. 1:**
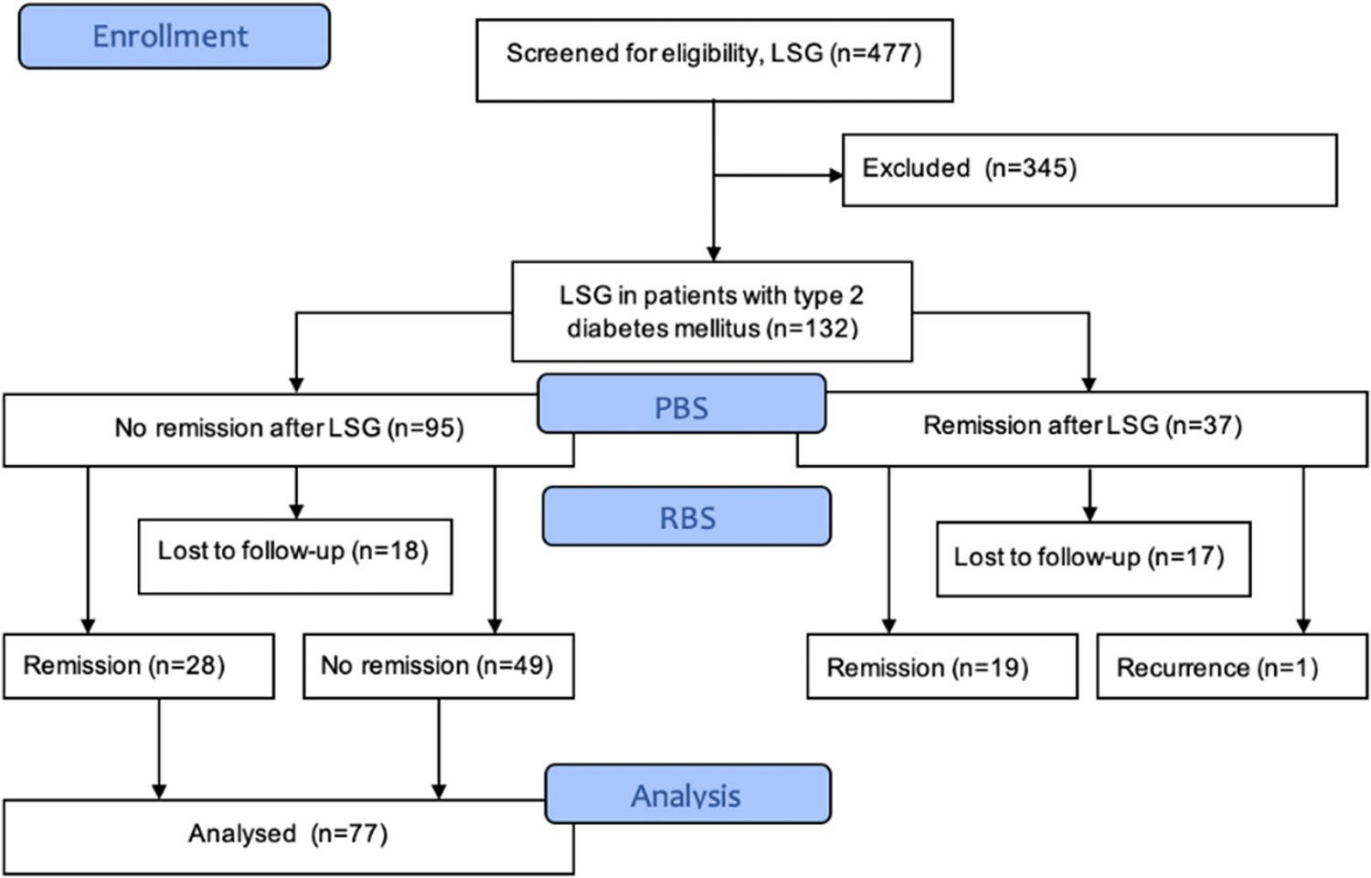
Patients flow-chart

Patients flow through the study is shown in Fig. 1. As presented in Fig. 1, 28% of 132 (37) had T2D remission after primary bariatric surgery. After RBS the T2D recurrence was observed in 1 patient, 19/37 sustained remission, while 17/37 patients were lost to follow-up. 57 females and 20 males were included in the study. Mean age was 51.0 ± 9.9 years. 28 patients (36.4%) had remission of T2D after RBS and were included in group 1, while 49 patients did not have remission of T2D and were analyzed in group 2.

Table 1 demonstrates general characteristics of the study groups prior to the LSG. Patients did not differ in terms of gender (*p* = 0.351), age (*p* = 0.358), maximal preoperative BMI (*p* = 0.312). 75% of patients in group 1 were treated with oral anti-diabetic medications, as compared to 63.4% in Group 2 (*p* = 0.793). Insulin injections were administered in 27.8% of patients from Group 1 and 26.5% of Group 2 (*p* = 0.558).

**Table 1:** General characteristics

	Group 1 – Remission of T2D after RBS	Group 2 – no remission of T2D after RBS	*P*-value
*n* (%)	28 (36.4%)	49 (63.6%)	n/a
Male/female, *n* (%)	9/19 (32.1%/67.9%)	11/38 (22.4%/77.6%)	0.351
Mean age, years ± SD	52.4 ± 10.0	50.2 ± 9.9	0.358
Median maximal BMI, kg/m^2^ (IQR)	51.2 (46.5–59.3)	50.0 (44.9–54.2)	0.312
Median BMI before primary procedure, kg/m^2^ (IQR)	47.2 (42.0–52.5)	47.0 (43.2–53.3)	0.641
Duration of obesity, *n* (%)			
< 5 years	1 (3.6%)	4 (8.2%)	0.848
5–15 years	11 (22.9%)	18 (36.7%)	
> 15 years	16 (33.3%)	27 (55.1%)	
Smoking, *n* (%)	4 (14.3%)	8 (16.3%)	0.555
Alcohol consumption, *n* (%)	5 (17.9%)	12 (24.5%)	0.915
NSAID or anticoagulation > once a week, *n* (%)	5 (17.9%)	5 (10.2%)	0.280
Oral anti-diabetic medications before PBS, *n* (%)	21 (75%)	31 (63.4%)	0.793
Insulin treatment before PBS, *n* (%)	8 (28.6%)	13 (26.5%)	0.558
Hypertension, *n* (%)	22 (78.6%)	40 (81.6%)	0.978
Asthma, obstructive sleep apnea, chronic obstructive pulmonary disease, *n* (%)	4 (14.3%)	6 (12.2%)	0.528
Prior gastric balloon treatment, *n* (%)	6 (21.4%)	3 (6.1%)	0.065

*IQR* inter-quartile range, *BMI* body mass index, *NSAID* non-steroid anti-inflammatory drugs

Table 2 shows weight loss outcomes after the primary bariatric procedure and reasons for revisional procedure. Median lowest BMI after primary LSG in Group 1 was 36.4 (34.0–42.1) kg/m^2^, while in Group 2 – 37.0 (32.8–39.9), (*p* = 0.941). Maximal BMI loss (*p* = 0.412) and %EBMIL (*p* = 0.388) did not significantly differ between groups. Time interval between primary LSG and RBS was significantly shorter in Group 1 than in Group 2 [1 (1–4) year vs. 3 (2–4) years, *p* = 0.023]. The most common reason for RBS in Group 1 was insufficient weight loss (< 50% EWL) or failure of remission of obesity related co-morbidities—15 patients (53.5%). While in the Group 2 it was weight regain (return to the original body weight after an initial successful weight loss)- 29 patients (59.2%). No significant differences in distributions were observed. Majority of patients in both groups had RBS performed in the same center as primary procedure (75% vs. 79.6%, *p* = 0.640). What is important, the median pre-RBS BMI was comparable in both groups, Group 1 – 42.1 (36.8–45.4) kg/m^2^ vs. Group 2 – 42.2 (37.0–46.6), *p* = 0.687, Table 2.

**Table 2:** Primary bariatric treatment and qualification for RBS details

	Group 1 – Remission of T2D after RBS	Group 2 – no remission of T2D after RBS	*P*-value
*n* (%)	28 (36.4%)	49 (63.6%)	n/a
Median lowest BMI after primary procedure, kg/m^2^ (IQR)	36.4 (34.0–42.1)	37.0 (32.8–39.9)	0.941
Median maximal BMI loss after primary procedure, kg/m^2^ (IQR)	−9.4 (−14.7; −6.7)	−11.4 (−17.6; −6.9)	0.412
Median %EBMIL after primary procedure (IQR)	52.9% (45.0%)-68.2%)	51.9% (36.3%-65.2%)	0.388
Median interval between primary procedure and RBS, years (IQR)	1 (1–4)	3 (2–4)	**0.023**
RBS due to complications of primary procedure, n (%)	2 (7.1%)	6 (12.2%)	0.703
RBS due to weight regain, n (%)	13 (46.4%)	29 (59.2%)	0.280
RBS due to insufficient weight loss or control of obesity co-morbidities, n (%)	15 (53.5%)	16 (32.7%)	0.072
Treatment continued in center that performed primary procedure, n (%)	21 (75%)	39 (79.6%)	0.640
BMI pre-RBS, kg/m^2^ (IQR)	42.1 (36.8–45.4)	42.2 (37.0–46.6)	0.687
Median difference in BMI pre-RBS and lowest after primary procedure, kg/m^2^ (IQR)	+ 2.8 (0–7.1)	+ 3.9 (0–7.4)	0.317

*IQR* inter-quartile range, *BMI* body mass index, *RBS* re-do bariatric procedure

Table 3 presents 1-year follow-up results after RBS. There was 1 (3.6%)re-SG in Group 1, while 8 (16.3%) in Group 2. 1 SASIwas performed in first group (3.6%), while 3 (6.1%) in the second. RYGB was done in 4 (14.3%) in Group 1, while in 15 (30.6%) in Group 2. The most commonly performed procedure in both groups was OAGB, Group 1—22 (78.6%) cases, Group 2—23 (46.9%) cases.

**Table 3:** Follow-up after RBS

		Group 1 – Remission of T2D after RBS	Group 2 – no remission of T2D after RBS	*P*-value
*n* (%)		28 (36.4%)	49 (63.6%)	n/a
Type of RBS, *n* (%)	re-SG	1 (3.6%)	8 (16.3%)	0.053
	SASI	1 (3.6%)	3 (6.1%)	
	RYGB	4 (14.3%)	15 (30.6%)	
	OAGB	22 (78.6%)	23 (46.9%)	
Median BMI after RBS, kg/m^2^ (IQR)		31.4 (28.1–36.4)	35.5 (31.9–37.5)	**0.024**
Median %EBMIL (IQR)		73.8% (65.2%-83.5%)	62.7% (50.2%–73.4%)	**0.001**
Median %EBMIL from BMI before RBS (IQR)		53.7% (39.3%–71.3%)	35.4% (20.9–56.3%)	**0.022**
Remission of hypertension, *n* (%)		8 (36.4%)	4 (10.0%)	**0.044**
Perioperative and postoperative morbidity of RBS, *n* (%)		4 (14.3%)	11 (22.5%)	0.552
Clavien-Dindo classification of peri- and postoperative morbidity of RBS, n	IV	1	0	n/a
	IIIB	1	3	
	IIIA	0	1	
	II	2	7	

*RBS* re-do bariatric procedure, *%EBMIL* percentage of excess BMI loss, *re-SG* re-do sleeve gastrectomy, *SASI* single anastomosis stomach-ileal bypass with sleeve, *RYGB* Roux-en-Y gastric bypass, *OAGB* one-anastomosis gastric bypass

**Fig. 2:**
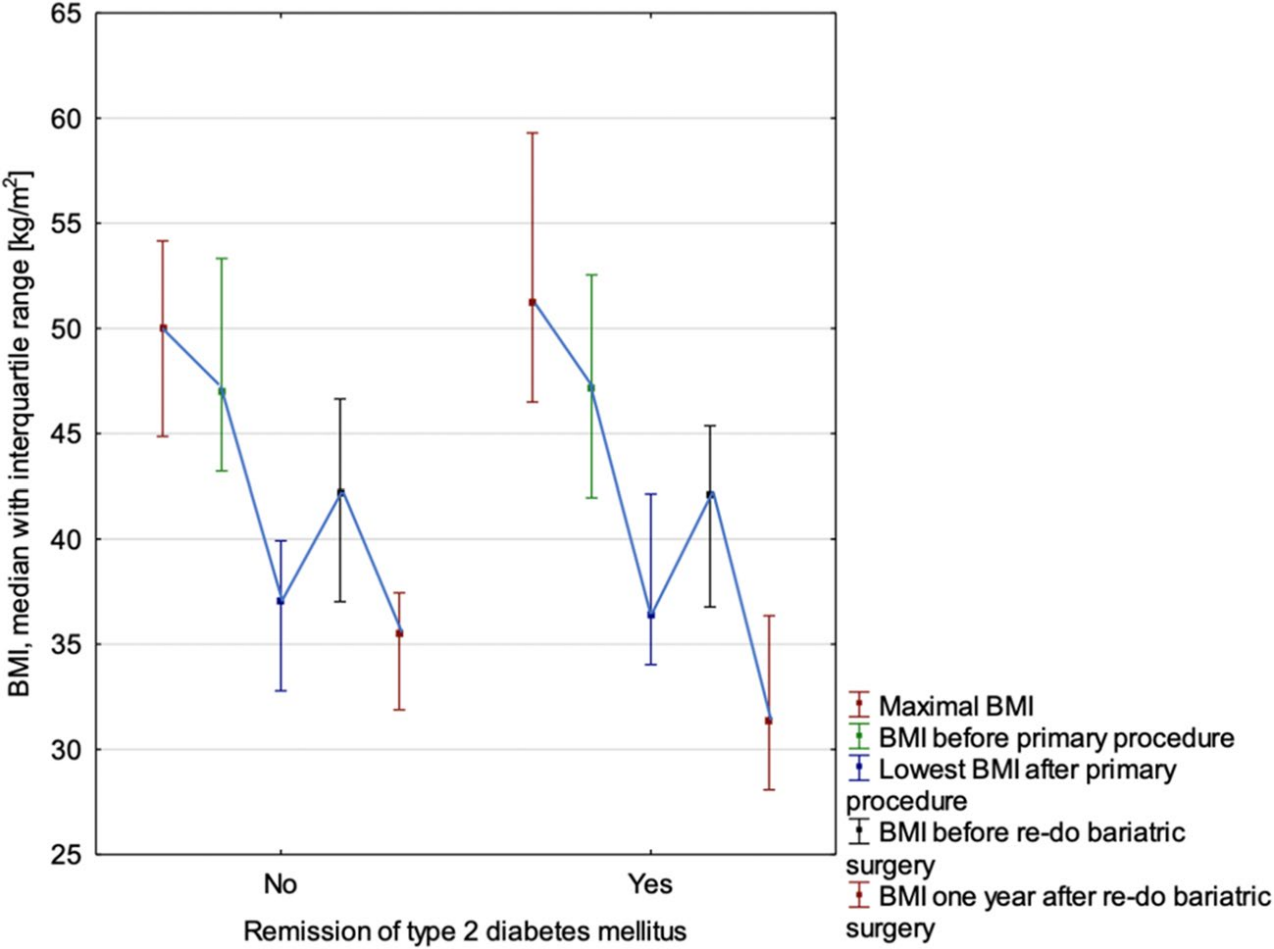
Changes in body mass index depending on remission type 2 diabetes mellitus one year after re-do bariatric surgery

Group 1 had significantly lower median BMI after RBS than Group 2 – 31.4 (28.1–36.4) vs. 35.5 (31.9–37.5) kg/m^2^, *p* = 0.024. Similarly, median %EBMIL – 73.8% (65.2%-83.5%) vs. 62.7% (50.2%-73.4%), *p* = 0.001, and median %EBMIL from BMI before RBS –53.7% (39.3%-71.3%) vs. 35.4% (20.9–56.3%), *p* = 0.022. Remission of hypertension was significantly more often observed in Group 1 than 2 (36.4% vs. 10.0%, *p* = 0.044). Perioperative and postoperative morbidity of RBS did not significantly differ between groups (*p* = 0.552). Changes in BMI in patients in both groups are presented in Fig. 2.

Morbidity of RBS one year after procedures did not differ groups significantly, 4 (14.3%) cases in Group 1 and 11 (22.5%) in Group 2 (*p* = 0.552). Morbidity in Group 1 included – 1 case of anemia and nutrition deficiencies; 2 cases of gastrointestinal leakage; 1 case of persistent vomiting and GERD. Morbidity in Group 2 was: 2 cases of renal failure requiring dialysis; 3 cases of anemia; 1 case of small intestine perforation,; 2 cases of severe GERD with esophagitis; 1 case of dysphagia; 1 case of surgical site infection and 1 case of Petersen's space hernia.

Table 4 shows results of univariate logistic regression model of all available factors potentially influencing remission of T2D after RBS. BMI after RBS and %EBMIL had significant influence on OR of T2D remission. Due to the fact that these variables are related, a multivariate regression was not possible. Next, the ROC curve analyses were performed. Significant cut-off point for BMI was set at 33 kg/m^2^ (AUC 0.66, 95%CI 0.53–0.78, *p* = 0.017), while for %EBMIL at 60.7% (AUC 0.72, 95%CI 0.61–0.84, *p* < 0.001). BMI after RBS lower than 33 kg/m^2^ resulted in 3.39 times higher odds ratio for T2D remission (95%CI 1.28–8.95, *p* = 0.014). %EBMIL greater than 60.7% resulted in 12.48 times higher OR for T2D remission (95%CI 2.67–58.42, *p* = 0.001).

**Table 4:** Univariate logistic regression analyses

	OR	95%CI	*p*-value
Female	0.61	0.22–1.73	0.353
Age	1.02	0.98–1.07	0.353
Maximal BMI	1.02	0.97–1.07	0.468
BMI before primary procedure	0.98	0.93–1.04	0.552
Duration of obesity			
< 5 years	ref		
5–15 years	2.00	0.18–22.06	0.571
> 15 years	1.83	0.17–19.32	0.617
Smoking	0.87	0.24–3.20	0.834
Alcohol consumption	0.75	0.21–2.76	0.669
NSAID or anticoagulation > once a week	2.05	0.46–9.11	0.344
Oral anti-diabetic medications	1.32	0.46–3.78	0.601
Insulin treatment	0.60	0.19–1.91	0.390
Hypertension	0.83	0.26–2.62	0.744
Asthma, obstructive sleep apnea, chronic obstructive pulmonary disease	1.19	0.31–4.66	0.798
Prior gastric balloon treatment	4.18	0.96–18.30	0.058
Maximal BMI loss after primary procedure	1.00	0.94–1.06	0.893
%EBMIL after primary procedure	1.01	0.99–1.03	0.522
Improved control of type 2 diabetes mellitus	1.29	0.48–3.47	0.615
Remission of hypertension	0.56	0.05–5.69	0.621
Improved control of hypertension	1.96	0.63–6.13	0.245
Interval between primary procedure and RBS	0.90	0.71–1.11	0.317
RBS due to complications of primary procedure	0.55	0.10–2.94	0.485
RBS due to weight regain	0.60	0.23–1.53	0.281
RBS due to insufficient weight loss or control of obesity co-morbidities	2.38	0.92–6.17	0.075
Treatment continued in center that performed primary procedure	0.77	0.26–2.32	0.641
BMI pre-RBS	0.98	0.92–1.04	0.492
Difference in BMI pre-RBS and lowest after primary procedure	0.96	0.88–1.05	0.399
BMI after RBS	0.91	0.82–0.99	**0.044**
BMI after RBS < 33 kg/m^2^	3.39	1.28–8.95	**0.014**
%EBMIL	1.04	1.01–1.08	**0.006**
%EBMIL > 60.7%	12.48	2.67–58.42	**0.001**
%EBMIL from BMI before RBS	1.01	0.99–1.03	0.101
Types of RBS			
re-SG	Ref		
SASI	2.67	0.12–57.62	0.532
RYGB	2.13	0.20–22.45	0.528
OAGB	7.65	0.88–66.32	0.065
Re-SG vs. malabsorptive	5.27	0.62–44.55	0.127
Perioperative and postoperative morbidity	0.58	0.16–2.02	0.388

*OR* odds ratio, *95%CI* 95% confidence interval, *RBS* re-do bariatric procedure, *%EBMIL* percentage of excess BMI loss, *re-SG* re-do sleeve gastrectomy, *SASI* single anastomosis stomach-ileal bypass with sleeve, *RYGB* Roux-en-Y gastric bypass, *OAGB* one-anastomosis gastric bypass

Table 5 set together rates of T2D remission after separately presented RBS depending of year, when RBS were performed.

**Table 5:** Patient reported impact on physical, social and psychological well-being after emergency laparotomy

*Physical symptoms*	*Social impact*	*Psychological impact*
Reduced walking distance	Can't relax when out socialising	Less optimistic about future
Altered bowel habit	Carrying stoma supplies	Feel worse about themselves
Stoma related issues, for example, parastomal herniae, flatus, leakage	Don’t go out as can't control what they eat	Feel more self-conscious
Fatigue/lack of energy	Need to know where bathroom is	Embarassment due to stoma and lack of control
Chronic pain	Attend fewer social activities	Self-confidence affected
Attend doctor more often		Less confident about job opportunities
Chronic infections		
Take more medications		
Cosmetic - scars and abdominal wall weakness		

## Discussion

There is a scarcity of research analyzing the factors that are increasing odds for T2D remission after RBS following insufficient metabolic outcome of primary LSG. Our retrospective cohort study aimed to analyze factors significantly increasing odds for T2D remission after RBS following primary LSG. The first finding was that time interval between primary LSG and RBS was significantly shorter in group with remission of T2D than in the group without remission. Median time interval was 1 (1–4) year in the group with remission vs. 3 (2–4) years in group without remission. Moreover, out of all factors potentially contributing to remission of T2D the only and significant was weight loss. BMI after RBS lower than 33 kg/m^2^ resulted in 3.39 times higher odds ratio for T2D remission, while %EBMIL greater than 60.7% resulted in 12.48 times higher OR for T2D remission.

In the STAMPEDE trial, one of the largest trials which enrolled 150 patients, the complete diabetes remission (glycated hemoglobin [HbA1c] < 6.0% without any medication) was observed in 42% patients one year after laparoscopic Roux-en-Y gastric bypass (LRYGB) and 27% after LSG [24]. At 3 years, 35% of LRYGB and 20% of LSG patients maintained diabetes remission [22].

21 LRYGB and 13 LSG patients that initially achieved remission after one year, 38% and 46% of them had recurrence of the disease after 3 years, despite only mild weight regain. Similar numbers were observed in patients flow through our study. Out of 132 patients after LSG with T2D initially, 28% had diabetes remission after primary bariatric surgery (PBS). After re-do surgery T2D recurrence was observed in 1 patient, other 19 sustained remission, and 17 patients were lost to follow-up.

However, based on recent published works, LSG does not appear to be significantly inferior to LRYGB at medium-term follow-up. This is reflected in some guidelines that are equating LSG and LRYGB in terms of metabolic outcomes [23, 24].

In our study, we analyzed 77 patients without remission of T2D after primary bariatric surgery. 36.4% of patients achieved remission after RBS at one-year follow-up. T2D remission rates after RBS performed after LSG varies between 11 and 67% [25–32]. In 7 studies that reported conversion of LSG to LRYGB 37 patients (18%) had residual diabetes prior to conversion. Reoperation added 9–26% of decrease in BMI, and improved T2D in 62% of patients [25–32]. Another 3 papers reported conversion to duodenal switch that added 26–30% BMI loss of pre-revision BMI, and 79% of the 17 patients had improvement of their T2D [27, 33, 34].

Indications for re-do surgery after LSG include predominantly weight regain, insufficient weight loss or control of obesity-related comorbidities, sleeve stenosis, and gastroesophageal reflux disease. Regardless of chosen option, the time for RBS seems significant. Shorter time intervals between primary and re-do surgery were observed more often in patients with remission of T2D after RBS in our cohort. However, that was insignificant factor for OR of DM2 remission in univariate logistic regression model. We did not found rationale for performing RBS earlier, but data in literature suggest strongly, that duration of T2D is the most probable, independent preoperative predictor of early diabetes relapse either no remission in patients with T2D undergoing metabolic surgery [35].

RBS options are re-sleeve gastrectomy or conversion to either LRYGB, SADI, SASI, OAGB or duodenal switch. Regretfully we failed to find literature providing comparison of those approaches and results in T2D remission after re-do surgeries. Similarly, factors that promote remission of T2D. Neverthless, the literature shows that even modest weight loss of 5–10% can significantly improve metabolic profile [35–38]. Meta-analysis on converting SG, laparoscopic adjustable gastric banding, and vertical banding gastroplasty to OAGB provided data on the effects of conversion on T2D with a range of remission up to 65–78% during the first 5 years [39]. Meta-analysis by Jie et al. investigated revisions after vertical banded gastroplasty in 462 (29.0%) patients, adjustable gastric banding in 434 (27.2%) patients, and SG in 432 (27.1%) patients [40]. Ninety-two percent of patients achieved improvement of T2D following revisional surgery. In subgroup analysis by type of RBS, similar rates of improvement were seen after rSG (98%) and RYGB (94%). For remission rates however, RYGB (62%) performed significantly better than r-SG (33%). Many postulate that the malabsorptive function of duodenal exclusion and benefits of gut hormones changes in small intestine bypasses are reason for that those should be chosen for RBS [41]. Another single center studies provide remission rates of T2D after RBS of SG as follows: 100% SASI, 85–100% after revisional OAGB, 60% after revisional RYGB, 88% after SADI [42–45]. Still, we lack firm evidence for procedure of choice in case of SG cases that need RBS for curative effect on T2D, what causes, that further research in this field is needed.

## Limitations

Study has limitations typical for a multicenter retrospective cohort studies. Another limitation is the definition for T2D remission that may vary between guidelines of bariatric and/or diabetologists associations and may cause difficulties to compare our results with other studies. Changes in overall treatment and procedure selection during 10-years study period in 12 centers are inevitably adding bias to study results. Also, the loss to follow-up at one-year in study group (18 patients) seems to be high. Variability of revisional procedures is adding bias, thus, the interpretation of the results must be done very carefully. Regretfully, the duration of T2D and its complications were not recorded in registry and registered follow-up in the participating centers that was accessible for investigation was 12 months after RBS.

## Conclusions

Shorter time interval between LSG and RBS is associated with increased odds for remission of T2D after revisional procedure. Significant loss of excess weight seems to be the most crucial factor for T2D remission.

## Data Availability

The data that support the findings of this study are available from the corresponding author upon reasonable request.
